# Developing a cognitive model of solid geometry based on Interpretive Structural Modeling method

**DOI:** 10.1016/j.heliyon.2024.e27063

**Published:** 2024-02-24

**Authors:** Heyang Zhang, Xiaopeng Wu, Mingyue Ju

**Affiliations:** a School of Mathematics and Statistics, Northeast Normal University, Changchun, China; b Faculty of Education, Northeast Normal University, Changchun, China; c Faculty of Education, The University of Hong Kong, Hong kong, China

**Keywords:** Online learning, Adaptive learning system, Cognitive model, Interpretive structural modeling (ISM), Mathematics education

## Abstract

With the advancement of science and artificial intelligence, education is experiencing significant innovation. The adaptive learning system is emerging as a promising approach to achieving personalized learning. The cognitive model plays a crucial role as the fundamental rationale behind the adaptive learning system. Currently, there is no uniform and highly operational method for constructing cognitive models. This study adopts Interpretive Structural Modeling (ISM) as the foundational approach for constructing a cognitive model of solid geometry. Based on literature and expert opinions, 17 cognitive attributes of high school solid geometry were identified. Subsequently, a questionnaire survey involving 40 experts was conducted to establish the contextual relationships among these attributes. Applying the ISM method resulted in a seven-level model. This model was then revised based on expert opinions to create the final cognitive model, revealing three primary paths within the domain of high school solid geometry.

This paper contends that the use of the ISM method for constructing cognitive models is effective and objective. The resulting cognitive model unveils the content structure of high school solid geometry, and provides an innovative perspective on the construction of cognitive models.

## 1 Introduction

With the rapid advancement of science and technology, the utilization of artificial intelligence and big data analysis in education is becoming increasingly prevalent. Under this background, online learning is challenging the status of traditional teaching and becoming an important way of learning [1]. Personalized learning is not only the ultimate objective of online learning but also the primary challenge it faces. To achieve this goal, educators proposed the terminology of adaptive learning system. Adaptive learning system is an emerging educational service system that utilizes various learning algorithms, such as artificial intelligence and machine learning to personalize students' learning experiences [2]. It can accurately evaluate the learning status of a large number of students, diagnose their learning deficiencies, and provide personalized learning resources for students [3], thereby promoting teaching accurately and effectively. Numerous studies have shown that adaptive learning systems have a positive effect on students’ learning [4,5].

Cognitive diagnostic assessment (CDA) is one theoretical basis for the adaptive learning system to achieve personalized learning [6]. The operation of CDA requires the development of a model or theory that describes the knowledge and skills hypothesized to be involved in test performance [7]. This model or theory is referred to as cognitive model [8,9]. Based on the cognitive model, CDA applies cognitive science and statistical models to infer students’ mastery of cognitive skills, generate fine-grained reports, and provide meaningful feedback [7]. In this way, a CDA-based adaptive learning system can achieve the goal of personalized learning. Through human-computer interaction, the deficiencies of students can be detected, and targeted teaching interventions can be carried out for them.

The objectivity, effectiveness, and operability of the cognitive model are crucial throughout the cognitive diagnosis process. Leighton et al. noted that the identification of the cognitive model of the test performance is crucial because it is the basis for predicting the categories of student performance and for inferring examinees’ cognitive competencies [9]. A high-quality cognitive model can even serve as a template for automatic test generation in the adaptive learning system [10]. The construction of cognitive model can be a challenging issue. Leighton et al. noted that the cognitive theories of performance are not always easily applied to assessment purposes [11]. The cognitive model contains a variety of individual knowledge and skills, and the hierarchical relationships between them are difficult to be determined consistently and objectively [12]. There is no uniform method for constructing cognitive models, and the existing methods have problems of being time-consuming or inaccurate [13].

To address the difficulties in dealing with complex issues or systems, Warfield developed a methodology that uses systematic application of some elementary notions of graph theory and Boolean algebra to construct directed graph (a representation of the hierarchical structure of the system) [14,15]. This approach has gained increasing popularity among researchers across various fields of study [16,17]. The ISM method is theoretically applicable in the construction of cognitive models, and has been tentatively applied by Sun, Wu and Xu, who built a mathematical cognitive model by ISM [18]. However, the cognitive model they built is not based on a specific mathematical knowledge domain. It is more meaningful to establish cognitive models grounded in specific knowledge, which can be directly applied to adaptive learning systems. Therefore, it still needs more in-depth attempts to use the ISM to construct cognitive models.

## 2 Literature review

### 2.1 Research on cognitive model and its construction

Traditional test practices place more emphasis on statistical technique than on the psychology of the construct being measured [19]. The test theories of traditional assessments are aimed at estimating a person's location on an underlying latent variable, such as a true score in classical test theory (CTT) and a latent trait in unidimensional Item Response Theory (IRT) [7]. The scores derived from traditional CTT or IRT approaches provide only general information to guide specific instructional decisions [20]. Whereas CDA makes it explicit the processes and knowledge structure a performer in the test domain would use by the theory of cognitive model [7]. It can provide students with information on whether they have mastered each skill within a specific group of skills, presented in the form of examinee classification [21,22]. Cognitive model is defined in education research as “a simplified description of human problem solving on standardized educational tasks, which helps to characterize the knowledge and skills students at different levels of learning have acquired and to facilitate the explanation and prediction of students' performance” (p. 6) [9]. The knowledge and skills here are called cognitive attributes. Cognitive attribute is the cornerstone of cognitive diagnostic assessment [23]. The definition of cognitive attributes is not uniform. For example, Leighton and Gierl believe that attributes are operational skills and knowledge structures needed to complete a task [9,11]. Dogan and Tatsuoka believe that attributes are the procedures, skills, processes, strategies and knowledge necessary to complete a task [24]. There is no uniform standard for the types of attributes. In the early years, attributes were mainly rules applied in problem-solving. For example, Tatsuoka notes that the cognitive process of students doing signed subtraction is as follows: first, change the sign of subtraction in brackets, and then convert subtraction to an addition problem [25]. However, as attributes are utilized across a diverse range of contexts, they tend to become more generalized [26]. In the study of Dogan and Tatsuoka, they classified attributes into three categories: content attribute, process attribute and skill attribute [24]. For instance, in their research, the content attributes encompass “Basic concepts, properties and operations in whole numbers and integers,” the process attributes involve “Translate/formulate equations and expressions to solve a problem,” and the skill attributes include “Using proportional reasoning”. Cognitive research suggest that cognitive skills do not operate in isolation but belong to a network of interrelated processes [27]. Leighton et al. believe that the cognitive attributes should be organized hierarchically to clarify the psychological ordering among the attributes required to solve a test problem. In the hierarchies of cognitive attributes, an examinee is not expected to possess the latter attribute unless the former attribute is also present [11].

The effectiveness and operability of cognitive models determine whether a cognitive diagnostic assessment is effective [28]. Because of that, the construction of cognitive models is particularly important. The construction of a cognitive model is accomplished by examining the knowledge, operations, and strategies used by individuals in the process of problem-solving [8]. Generally, there are two ways to construct cognitive models [13,29]. 1. Based on experts. Establish a cognitive model through analysis of theories and literature, and then validate it through expert interviews to revise the cognitive model. 2. Based on students. Because cognitive models focus on problem-solving, which is a human information processing process, it is very suitable for using verbal reports to construct the cognitive model. This approach requires students to engage in “think aloud” activities when solving problems, in order to determine the knowledge and cognitive skills students adopt when facing tasks. There is no unified method for constructing cognitive models, and existing methods are inevitably influenced by the understanding of subjects. These methods all have deficiencies in objectivity and consistency [12].

### 2.2 Research on interpretive structural modeling

When encountering a complex system, people often need to clarify the relationships among the factors within the system. It is always the case that people can clarify the relationships between each pair of factors in the system, but it is difficult to have a comprehensive understanding of the relationship among all factors in the entire system. Interpretive Structural Modeling (ISM) is a way to solve this type of problem [14]. The ISM method transforms unclear, poorly articulated systems into visible and ordered models [30].

ISM offers a variety of advantages. In the ISM method, the determination of the contextual relationship between factors is based on the opinions of expert groups, so the model naturally has the characteristic of interpretability [31]. Compared to Delphi and structural equation modeling (SEM), ISM method has its own advantages [32]. The Delphi method is a structured technique used for forecasting information in a wide range of fields. It follows a series of steps to develop a consensus among a group of experts. However, it is very difficult to collect an adequate number of questionnaires from busy individuals [33]. The SEM approach has the capability of statistically testing an already-developed model whereas ISM has the capability to develop an initial model [32]. ISM has been widely applied in many practical production fields, such as green supply chain management [34], productivity improvement [35] and reverse logistics [36]. In recent years, the application of ISM in the education research field has become increasingly widespread, such as in curriculum construction [37], and graduate employment [38]. Cognitive model is a hierarchical structure composed of cognitive attributes, and the construction of a cognitive model involves the process of clarifying the hierarchical relationships between complex cognitive attributes. The functions and advantages of ISM make it a very suitable method for the construction of cognitive models. The deep integration of ISM and cognitive model construction is a new field worthy of in-depth research. Research on the construction of cognitive models in specific knowledge fields by ISM has important practical value for adaptive learning systems and online knowledge graph systems.

### 2.3 Research on solid geometry

Geometry is an important component of the discipline of mathematics. In America, the Common Core State Standards for Mathematics (2010) state that geometry in K-12 education can be divided into two major fields: synthetic geometry (without coordinates) and analytical geometry (with coordinates) [39]. From a global perspective, TIMSS (Trends in International Mathematics and Science Study) has great authority in curriculum research. The knowledge domain of TIMSS assessment is based on the curriculum documents from each of the participating countries [40]. TIMSS Advanced is a part of the TIMSS assessment, which studies students in their final year of secondary school [41]. In the TIMSS advanced mathematics framework, geometry covers two topics: non-coordinate and coordinate geometry, as well as trigonometry. The content of non-coordinate and coordinate geometry includes: 1. Use non-coordinate geometry to solve problems in two and three dimensions; 2. Use coordinate geometry to solve problems in two dimensions; 3. Apply the properties of vectors and their sums and differences to solve problems [42].

The learning process of geometry not only involves understanding and mastering geometric knowledge but also involves relevant skills and problem-solving strategies. The van Hieles proposed a model of the development of geometric thinking that identified five differentiated levels of thinking: identification, analysis, informal deduction, formal deduction, and rigor [43]. In 1991, Gutiérrez et al. conducted a study based on the van Hiele model, which divided the level of spatial geometry reasoning ability and explained the meaning of each level. They divided spatial reasoning into four aspects: Recognition: recognize and name solids (prisms, cones, pyramids, etc.); Analysis: identify the components of solids (faces, edges, etc.), and know the solids are bearers of their properties (parallelism, regularity, etc.); Non-formal deduction: students can logically classify families of solids (classes of prisms or rounded solids, regular polyhedra, etc.); Formal deduction: Students understand the roles of the different elements of an axiomatic system (axioms, definitions, undefined terms, and theorems) and can perform formal proofs [44]. Spatial ability is the ability to generate, retain, retrieve and transform visual images [45]. Unal et al. defined spatial ability as two aspects in a study of spatial thinking among pre-service teachers: spatial orientation and spatial visualization [46]. Spatial ability is extremely important for the learning of three-dimensional geometry. Only by understanding geometry from the perspective of spatial perception can two-dimensional images be viewed as three-dimensional in mind.

Solid geometry is an crucial part of mathematics and mathematics education. It is not only an important venue for cultivating students' intuitive imagination ability, but also plays a role in cultivating students’ logical reasoning literacy and axiomatic thinking [47,48]. At the same time, solid geometry is a complex learning field where multiple knowledge and skills are intertwined. Clarifying this complex system into a clear and hierarchical structure and establishing a comprehensive cognitive model are of great practical significance for better understanding student learning, constructing teaching sequences, and developing adaptive learning systems. So, the research questions of this paper are:

1. What are the cognitive attributes of high school solid geometry?
2. How to construct a cognitive model of high school solid geometry using ISM approach?

## 3 Steps involved in ISM methodology

**Fig. 1:**
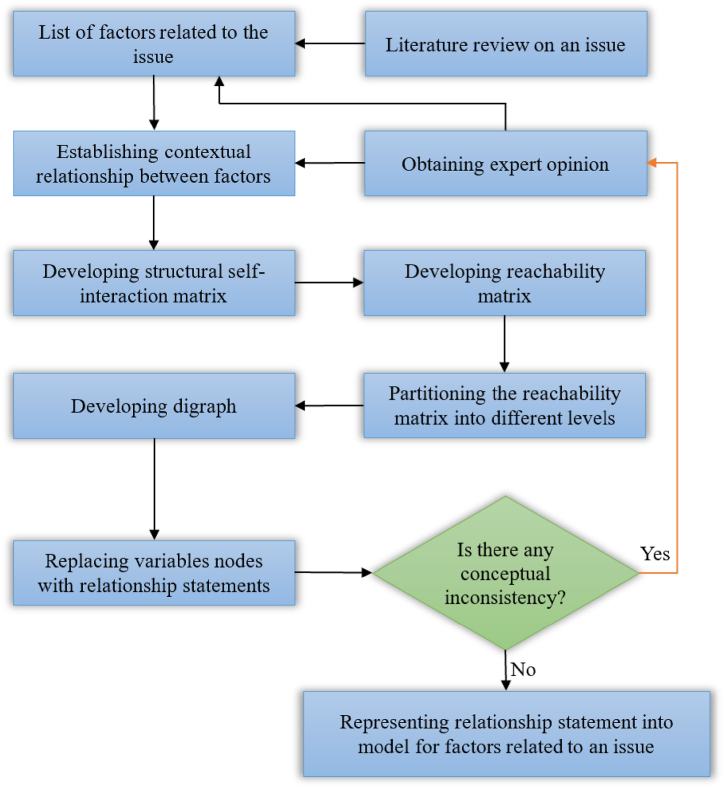
Flow diagram for implementing ISM methodology.

The steps that need to be followed when implementing ISM method are described in Fig. 1:

### 3.1 Factors determination

This step is the starting point of the ISM method. The identification of the factors that are relevant to the problem could be done by a survey or group problem-solving technique [15].

### 3.2 Structural self-interaction matrix

This step requires clarifying the contextual relationship among each pair of elements. ISM methodology suggests the use of expert opinions based on various management techniques such as brain storming, nominal group techniques and idea engineering in developing the contextual relationship among the variables [35,49]. The experts who are surveyed should possess expertise in the field where the problem located. The “leads to” or “influences” type of contextual relationship should be identified when analyzing the factors. The Structural Self-Interaction Matrix (SSIM) is composed of the relationships among factors. In the SSIM, the relationship between two factors (i and j) is represented by the four symbols V, A, X and O:

V indicates factor i will help to achieve factor j;
A indicates factor j will help to achieve factor i;
X indicates factor i and j will help to achieve each other;
O indicates factors i and j are unrelated.

Based on the contextual relationships, the SSIM can be developed [15].

### 3.3 Reachability matrix

The initial reachability matrix can be converted by the SSIM matrix from last step. This could be done by substituting the symbols (V, A, X, and O) in the SSIM by 1s or 0s. In the initial reachability matrix, the relationship between factor i and factor j is represented by the entry *a*_*ij*_. When the factor i will help to achieve factor j, *a*_*ij*_ = 1; when the factor i has no direct influence on factor j, *a*_*ij*_ = 0.

After the preparation of the initial reachability matrix, computational programs such as Boolean algebra algorithms are utilized to convert the initial reachability matrix to the final reachability matrix [32].

### 3.4 Level partition

Starting from the reachability matrix, the reachability set and antecedent set of each factor should be derived. The reachability set of a particular factor consists of the factor itself and the other factor which it may impact, while the antecedent set consists of the factor itself and the other factor that may impact it. The intersection set of the reachability set and the antecedent set of all factors also needed to be derived [32]. If the reachability set of some factors is the same as the intersection set, assign the factor to the top-level of the system because they would not help achieve any other factors above their own level. Once all of the top-level factors are determined, they will be removed from consideration and the procedure above is repeated to find out the factors at the next level. The process is continued until the level of all factors is determined.

### 3.5 Digraph

In this procedure, the top-level factor is positioned at the top of the digraph and the second level factor is placed in the second position and so on. Linking all factors according to the levels yields the preliminary digraph (directed graph) composed of nodes and lines [30]. After removing the indirect links, the final digraph is obtained. The digraph is a visual representation of the factors and their interdependence [36]. After obtaining the digraph, it is necessary to check whether there are any parts of the obtained graph that need to be modified based on the actual situation of the problem in order to comply with reality.

As this study aims to apply the ISM method to the construction of cognitive models, the problem we need to focus on is the construction of cognitive models for high school solid geometry, and the related factors are various cognitive attributes in solid geometry.

## 4 cognitive model construction

### 4.1 Cognitive attributes determination

Based on the analysis of relevant research literature on geometry as well as the frameworks of TIMSS and Common Core State Standards for Mathematics in the literature review section, cognitive attributes of high school solid geometry can be preliminarily obtained. According to Unal et al.‘s view of spatial ability, some key attributes can be obtained: spatial visualization, geometric drawing [46]. According to Gutiérrez et al.'s framework, some key attributes can be obtained: recognize solids, define solids, geometric positional relationships, understand axiomatic system, and conduct deductive reasoning [44]. Through the analysis of the TIMSS advanced 2015 mathematics framework, some key attributes can be obtained: space coordinate system, understand spatial vectors and their operations, and conduct geometric modeling [42]. After summarizing and organizing these objects, this study determines 17 cognitive attributes. These attributes can theoretically cover the knowledge and skills that one should possess during high school solid geometry problem-solving. These attributes are summarized in Table 1:

**Table 1:** Cognitive attributes of high school solid geometry.

Attributes	Code	Description
Spatial Visualization	A1	Observe solids and form spatial perception, and be able to form the shape of solids in mind
Recognize solids	A2	Be able to identify columns, cones, balls, and their simple combinations in real life and in images
Define solids	A3	Define solids such as straight prism, oblique prism, parallelepiped, etc.
Classify solids	A4	Classify solids by their geometric properties
Geometric drawing	A5	Draw a visual representation of solids on a plane (sphere, cylinder, cone, prism, and their simple combinations)
Geometric positional relationships	A6	Understand and recognize the positional relationships between points, lines, and planes in three-dimensional space
Represent geometric propositions	A7	Use mathematical language to express geometric propositions, such as propositions about parallelism judgment
Axiomatic system	A8	Know that geometric postulates are self-evident facts and can use definitions and basic facts for logical deduction
Use geometric postulates	A9	Conduct mathematical reasoning using axioms and postulates in Euclidean geometry
Space coordinate system	A10	Understand the spatial Cartesian coordinate system and be able to draw a spatial Cartesian coordinate system on the paper
Represent geometric objects with coordinates	A11	Use coordinates to represent geometric objects such as points and lines
Space vector	A12	Know the definition of space vectors and be able to perform operations with space vector
Coordinate representation of vectors	A13	Use coordinates to represent the sum and the difference of space vectors
Represent geometric propositions with vectors	A14	Use vectors to represent lines parallel, surfaces parallel etc. in three-dimensional space
Select geometric problem-solving strategies	A15	Flexibly choose to use vectors, coordinate system, and other methods to solve geometric problems
Geometric modeling	A16	Use geometric knowledge to model geometric phenomena in the real world
Geometric deductive reasoning	A17	Use deductive reasoning and existing theorems as tools to verify geometric propositions

### 4.2 Structural self-interaction matrix development

After determining initial cognitive attributes, we clarified the contextual relationship between each pair of attributes through the opinions of experts in the field of mathematics curriculum and teaching. This study adopts the method of questionnaire survey through online questionnaire software: Questionnaire Star. This study involved 36 in-service high school teachers, 4 graduate students in mathematics education, and a total of 40 people as the survey subjects. We first introduced the meaning of each cognitive attributes and then asked the experts to judge the pairwise relationship between the 17 cognitive attributes. When more than half (>20) of the experts believe that the influence relationship exists, we will confirm the relation. The SSIM was formed according to expert opinions.

The SSIM is shown in Table 2:

**Table 2:** Structural self-interaction matrix.

V	V	V	V	V	V	V	V	V	O	V	V	V	V	V	V	A1
O	V	V	O	O	O	O	V	V	O	O	O	V	V	V	A2	
O	V	V	O	O	O	O	O	V	O	O	O	O	V	A3		
O	V	V	O	O	O	O	O	V	O	O	A	O	A4			
O	V	V	O	V	V	V	V	O	O	O	A	A5				
V	V	V	V	V	V	V	V	V	O	V	A6					
V	V	V	V	V	V	V	O	V	O	A7						
V	O	V	O	O	O	O	O	V	A8							
V	O	V	O	O	O	O	O	A9								
O	V	V	O	V	O	V	A10									
O	V	V	O	O	O	A11										
O	V	V	V	V	A12											
O	V	V	O	A13												
O	V	V	A14													
A	X	A15														
O	A16															
A17																

### 4.3 Reachability matrix development

The initial reachability matrix can be obtained directly from the SSIM above. After obtaining the initial reachability matrix, the final reachability matrix R was calculated through MATLAB R2019a software, and the obtained final reachability matrix is shown in (1).

(1) $$R=\left(\begin{array}{ccccccccccccccccc} 1 & 1 & 1 & 1 & 1 & 1 & 1 & 0 & 1 & 1 & 1 & 1 & 1 & 1 & 1 & 1 & 1\\ 0 & 1 & 1 & 1 & 1 & 0 & 0 & 0 & 1 & 1 & 1 & 1 & 1 & 1 & 1 & 1 & 1\\ 0 & 0 & 1 & 1 & 0 & 0 & 0 & 0 & 1 & 0 & 0 & 0 & 0 & 0 & 1 & 1 & 1\\ 0 & 0 & 0 & 1 & 0 & 0 & 0 & 0 & 1 & 0 & 0 & 0 & 0 & 0 & 1 & 1 & 1\\ 0 & 0 & 0 & 0 & 1 & 0 & 0 & 0 & 0 & 1 & 1 & 1 & 1 & 1 & 1 & 1 & 0\\ 0 & 0 & 0 & 1 & 1 & 1 & 1 & 0 & 1 & 1 & 1 & 1 & 1 & 1 & 1 & 1 & 1\\ 0 & 0 & 0 & 0 & 0 & 0 & 1 & 0 & 1 & 0 & 1 & 1 & 1 & 1 & 1 & 1 & 1\\ 0 & 0 & 0 & 0 & 0 & 0 & 0 & 1 & 1 & 0 & 0 & 0 & 0 & 0 & 1 & 1 & 1\\ 0 & 0 & 0 & 0 & 0 & 0 & 0 & 0 & 1 & 0 & 0 & 0 & 0 & 0 & 1 & 1 & 1\\ 0 & 0 & 0 & 0 & 0 & 0 & 0 & 0 & 0 & 1 & 1 & 0 & 1 & 0 & 1 & 1 & 0\\ 0 & 0 & 0 & 0 & 0 & 0 & 0 & 0 & 0 & 0 & 1 & 0 & 0 & 0 & 1 & 1 & 0\\ 0 & 0 & 0 & 0 & 0 & 0 & 0 & 0 & 0 & 0 & 0 & 1 & 1 & 1 & 1 & 1 & 0\\ 0 & 0 & 0 & 0 & 0 & 0 & 0 & 0 & 0 & 0 & 0 & 0 & 1 & 0 & 1 & 1 & 0\\ 0 & 0 & 0 & 0 & 0 & 0 & 0 & 0 & 0 & 0 & 0 & 0 & 0 & 1 & 1 & 1 & 0\\ 0 & 0 & 0 & 0 & 0 & 0 & 0 & 0 & 0 & 0 & 0 & 0 & 0 & 0 & 1 & 1 & 0\\ 0 & 0 & 0 & 0 & 0 & 0 & 0 & 0 & 0 & 0 & 0 & 0 & 0 & 0 & 1 & 1 & 0\\ 0 & 0 & 0 & 0 & 0 & 0 & 0 & 0 & 0 & 0 & 0 & 0 & 0 & 0 & 1 & 1 & 1 \end{array}\right)$$

### 4.4 Level partition

The first step is to derive the reachability set of each attribute. The reachability set of attribute i is equal to the set of attributes with the value of 1 in the i-th row of the final reachability matrix, denoted as *R (Ai)*; The second step is to derive the antecedent set of each attribute. The antecedent set of attribute i is equal to the set of attributes with the value of 1 in the i-th column of the final reachability matrix, denoted as *A (Ai)*; The third step is to intersect the reachability set and antecedent set of each attribute to derive the intersection set, denoted as *R (Ai)∩ A (Ai)*; When the elements in the reachability set of an attribute are the same as those of the intersection set, the element is extracted. The elements extracted in the first batch are the level 1 element set. After extracting the attributes of level 1, delete the extracted attributes of level 1 to obtain a new reachability matrix. Repeat the above steps for the extraction of level 2, and so on. The following Table 3 shows an example of the extracting process for level 1 attributes.

**Table 3:** The partition process of the first level of the cognitive model.

Attribute	*R (Ai)*	*A (Ai)*	*R (Ai)*∩*A (Ai)*	Level
A1	1,2,3,4,5,6,7,9,10,11,12,13,14,15,16,17	1	1	
A2	2,3,4,5,9,10,11,12,13,14,15,16,17	1,2	2	
A3	3,4,9,15,16,17	1,2,3	3	
A4	4,9,15,16,17	1,2,3,4,6	4	
A5	5,10,11,12,13,14,15,16	1,2,5,6	5	
A6	4,5,6,7,9,10,11,12,13,14,15,16,17	1,6	6	
A7	7,9,11,12,13,14,15,16,17	1,6,7	7	
A8	8,9,15,16,17	8	8	
A9	9,15,16,17	1,2,3,4,6,7,8,9	9	
A10	10,11,13,15,16	1,2,5,6,10	10	
A11	11,15,16	1,2,5,6,7,10,11	11	
A12	12,13,14,15,16	1,2,5,6,7,12	12	
A13	13,15,16	1,2,5,6,7,10,12,13	13	
A14	14,15,16	1,2,5,6,7,12,14	14	
A15	15,16	1,2,3,4,5,6,7,8,9,10,11,12,13,14,15,16,17	15,16	1
A16	15,16	1,2,3,4,5,6,7,8,9,10,11,12,13,14,15,16,17	15,16	1
A17	15,16,17	1,2,3,4,6,7,8,9,17	17	

Through the steps above, it can be concluded that the attributes in level 1 are: A15: Select geometric problem-solving strategies, A16: Geometric modeling; The attributes contained in level 2 are: A11: Represent geometric objects with coordinates, A13: Coordinate representation of vectors, A14: Represent geometric propositions with vectors, A17: Geometric deductive reasoning; The attributes contained in level 3 are: A9: Use geometric postulates, A10: Space coordinate system, A12: Space Vector; The attributes contained in level 4 are: A4: Classify solids, A5: Geometric drawing, A7: Represent geometric propositions, A8: Axiomatic system. The attributes contained in level 5 are: A3: Define solids, A6: Geometric positional relationships. The attribute contained in level 6 is: A2: Recognize solids. The attribute contained in level 7 is: A1: spatial visualization.

### 4.5 Cognitive model development

**Fig. 2:**
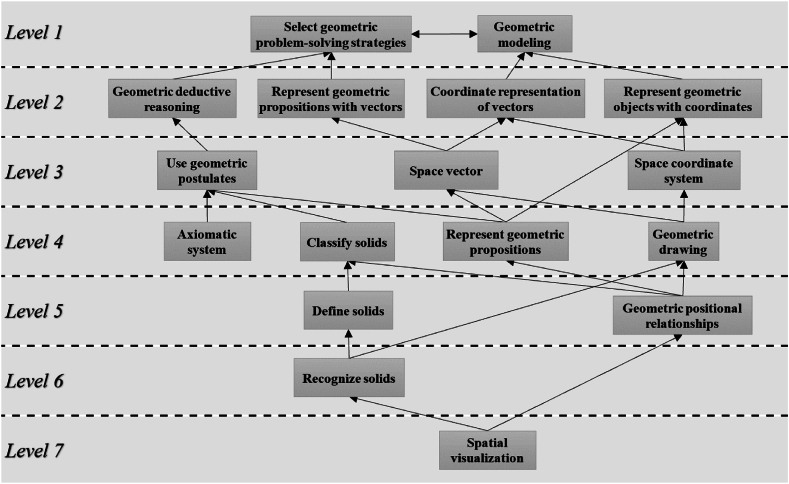
Preliminary digraph.

By applying the ISM method, a preliminary digraph can be obtained, which is shown in Fig. 2.

**Fig. 3:**
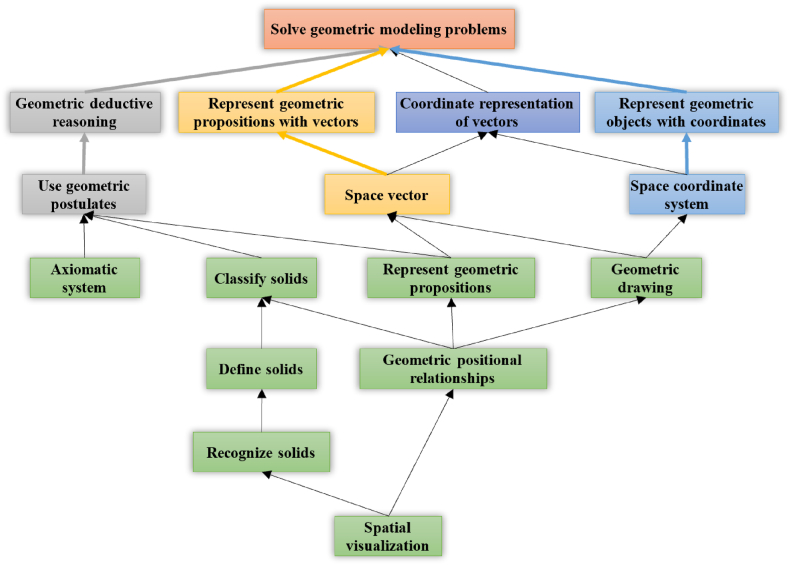
Final cognitive model.

Here, it is necessary to validate and revise the digraph based on the disciplinary nature and the properties of the cognitive model. We revised the ISM-based model through discussion with two curriculum experts. Because the influence relationship of the “Recognize solids” on the “Geometric drawing”, as well as the influence relationship of “Represent geometric propositions” on the “Represent geometric objects with coordinates” are not clearly reflected in the discipline of mathematics, these two lines are deleted. The influence relationship between “Select geometric problem-solving strategies” and “Geometric modeling” is mutual. These two attributes should be considered as one in the theory of cognitive diagnostic assessment. We combined the two attributes into one: “Solve geometric modeling problems.” Then the cognitive model obtained in this way conforms to the discipline of mathematics and the cognitive diagnostic assessment theory. The final cognitive model is shown in Fig. 3.

It can be seen that the cognitive model of high school solid geometry starts with spatial visualization, and goes through the process of recognizing and defining solids, as well as understanding geometric positional relationships. These basic attributes are the preliminary of key abilities such as classify solids, represent geometric propositions, and geometric drawing. The attributes mentioned above are the foundation for complex solid geometry problem-solving. After these knowledge and skills, the cognitive model can be divided into three main paths:

1. The first path is “use geometric postulates”→ “geometric deductive reasoning”, thereby solving geometric problems. This path only relies on deductive reasoning and can be considered within the field of synthetic geometry, which is the focus of traditional Euclidean geometry. This path can be named “synthetic path”.
2. The second path is “space vector” → “represent geometric propositions with vectors”. This path uses vectors as a tool for problem-solving. Vector is an important concept both in mathematics and science [50]. By utilizing vectors, students can effortlessly tackle geometric problems. This path can be named “vector path”.
3. The third path is “space coordinate system” → “represent geometric objects with coordinates”, thereby solving geometric problem. Coordinate system is another important tool for studying geometry. Since Descartes introduced coordinates into geometry, a new direction of geometric research using coordinates has emerged: analytical geometry [51]. In the high school curriculum, the ideas and methods in analytical geometry are also particularly important. This path can be named “coordinate path”.

There is also an attribute between “coordinate path” and “vector path”, which is “Coordinate representation of vectors”. It represents the combination of coordinate system and vector, which is also a powerful tool for solving geometric problems.

## 5 Discussion

With the advancement of science and technology, online learning systems such as adaptive learning system and online knowledge graph system are making significant progress. Both adaptive learning system and knowledge graph system require modeling of students’ cognitive processes [2]. Researchers need an efficient and time-saving method for the construction of cognitive models [13]. This study applied ISM method to construct a cognitive model of high school solid geometry. Based on expert opinions, further validation and improvement were conducted on the ISM-based model. We finally obtained a reasonable and informative cognitive model. This study further deepens the application of ISM method in the construction of cognitive model, and enriches practical experience in this field [18].

The cognitive model we obtained starts with the attribute of “spatial visualization” and goes through multiple paths to achieve the attribute of “Solve geometric modeling problems”, which comprehensively depicts various problem-solving processes in the field of solid geometry. In the van Hiele theory, geometric thinking is divided into five levels: identification, analysis, informal deduction, formal deduction and rigor [43]. To some extent, the cognitive model is consistent with van Hiele theory. “Spatial visualization”, “Geometric positional relationships” are located at the bottom of the model, which indicates that they have a driving effect on the upper-level attributes [52]. In order to foster higher-order problem-solving techniques, it is important to develop these basic skills in solid geometry teaching [46,53]. By clearly understanding students’ cognitive processes and adapting them to the most appropriate teaching activities, tasks, tools, interventions, and evaluation methods, students can gradually master the complex concepts. It can also be found that “Recognize solids” and “Define solids” are located in a relatively low position, indicating that it is necessary to use basic solids as the carrier when learning solid geometry [44]. The attributes such as “Classify solids”, “Represent geometric propositions”, and “Geometric drawing” are located in the middle part of the model, which are not only the results of learning basic geometry knowledge and skills but also play a central role in reaching the three problem-solving paths in the upper level of the model.

In the upper level of the final cognitive model, three problem-solving paths for high school solid geometry can be clearly identified: “synthetic path”, “vector path” and “coordinate path”. Synthetic geometry is a disciplinary method that uses logical deduction to solve problems in geometry [54]. In the cognitive model, this path is “Use geometric postulates” to “Geometric deductive reasoning”, which conforms to the paradigm of synthetic geometry. Analytic geometry is a disciplinary method that uses the algebra approach to solve geometric problems [55]. In the cognitive model, this path is through “Space coordinate system” to “Represent geometric objects with coordinates”. Vector is an important concept that holds an important position in both mathematics and physics [56]. In the model, the vector path is through the “Space vector” to “Represent geometric propositions with vectors”. Therefore, the cognitive model constructed based on ISM method clearly depicts the essence of solid geometry, which is in line with its disciplinary logic.

The construction of cognitive models has always been a challenge in the process of cognitive diagnosis assessment [11,12]. We always encounter the problem of strong subjectivity, which ultimately leads to the failure to obtain a unified model [12]. This successful attempt shows that this application of ISM method for constructing cognitive models can be generalized to other knowledge fields. In addition to geometry, there are also other core knowledge areas such as algebra, function, probability, and statistics in mathematics. This application can even be extended to other disciplines, such as science. However, there are also certain issues worth noticing when using ISM method to construct cognitive models. If the contextual relationships between factors are too complex, it will be very difficult to construct an effective cognitive model. In the initial ISM-based model in this study, the influence relationship between “Select geometric problem-solving strategies” and “Geometric modeling” is mutual. However, the mutual influence between attributes is meaningless in the theory of CDA. It can be concluded that the ISM method must be combined with expert verification to correct potential errors that may occur [15].

## 6 Conclusion and implications

This study applies the ISM method to the construction of cognitive model in a specific knowledge domain of mathematics and obtains an ISM-based model with 7 levels. After discussion with experts, a final cognitive model is obtained. In conclusion, this cognitive model possesses theoretical validity. This model is consistent with van Hiele theory, as well as the research within mathematics education [43,44,46]. Three problem-solving paths for high school solid geometry can be clearly identified from the model: “synthetic path”, “vector path” and “coordinate path”. It clearly explains the hierarchical structure of the discipline, so it has the potential to be applied directly to the construction of adaptive learning system and online knowledge graph system. When the cognitive model is applied, due to the strict hierarchical relationship among attributes, the calibration process of the attributes of test questions can be automated, achieving a time-saving and labor-saving effect [10].

This study offers a relatively objective and time-saving method to construct cognitive models compared to existing methods [13]. It is worth noting that the result should be revised and corrected by experts to ensure effectiveness. This method is worth generalizing to a certain extent. At present, the online knowledge graph system is also becoming increasingly popular in education [57]. The operation of online knowledge graph system also needs to model students’ cognitive processes in a certain knowledge field and match corresponding questions and learning resources based on the model. Our way of constructing cognitive models using the ISM method can also be applied to the development process of online knowledge graph system.

## 7 Limitations

This study applies ISM method to construct a cognitive model of high school solid geometry. There are some limitations in this study. Firstly, the attribute determination in this study was completed through the literature review and expert opinions. However, although it is less subjective than traditional methods, there is still subjectivity within, so further research is needed to verify whether these attributes are comprehensive and accurate in covering solid geometry. Secondly, while the model obtained in this study is based on specific knowledge content and possesses a certain degree of practicality, further analysis is necessary to ascertain its applicability in an adaptive learning system. The utility of the cognitive model obtained in this study can be further verified by using structural equation modeling and cognitive diagnostic assessment in future large-scale assessments.

## Funding

This work was supported by China Industry-University-Research Innovation Fund: Construction of an adaptive learning system for mathematical Cognitive diagnosis based on 3D Knowledge Graph (2022MU031).

## Ethics statement

This study was approved by the Ethics Committee of East China Normal University (protocol code HR663-2022, approved on November 11, 2022).

## Data availability statement

The datasets generated and/or analyzed during the current study are available from the corresponding author upon reasonable request.

## CRediT authorship contribution statement

**Heyang Zhang:** Writing – original draft. **Xiaopeng Wu:** Writing – review & editing, Supervision, Methodology, Funding acquisition, Conceptualization. **Mingyue Ju:** Writing – review & editing, Investigation.

## Declaration of competing interest

The authors declare that they have no known competing financial interests or personal relationships that could have appeared to influence the work reported in this paper.
