# Supplementary material for: Developing a cognitive model of solid geometry based on Interpretive Structural Modeling method
Source: Heliyon. 2024 Feb 24;10(5):e27063. doi: 10.1016/j.heliyon.2024.e27063 (PMC10943343; doi:10.1016/j.heliyon.2024.e27063)
Supplement: Multimedia component 1 [file mmc1.docx]

**Questionnaire on the Relationships of Cognitive Attributes in Solid Geometry**

1. Please choose your current occupation:

| ( ) High school mathematics teacher |
| --- |
| ( ) Graduate students in mathematics education |

2. Does “Spatial Visualization” require the following knowledge and skills?

(Click the options that you think are required)

| ( ) Recognize solids | ( ) Define solids |
| --- | --- |
| ( ) Classify solids | ( ) Geometric drawing |
| ( ) Geometric positional relationships | ( ) Represent geometric propositions |
| ( ) Axiomatic system | ( ) Use geometric postulates |
| ( ) Space coordinate system | ( ) Represent geometric objects with coordinates |
| ( ) Space vector | ( ) Coordinate representation of vectors |
| ( ) Represent geometric propositions with vectors | ( ) Select geometric problem-solving strategies |
| ( ) Geometric modeling | ( ) Geometric deductive reasoning |

3. Does “Recognize solids” require the following knowledge and skills?

(Check the options that you think are required)

| ( ) Spatial Visualization | ( ) Define solids |
| --- | --- |
| ( ) Classify solids | ( ) Geometric drawing |
| ( ) Geometric positional relationships | ( ) Represent geometric propositions |
| ( ) Axiomatic system | ( ) Use geometric postulates |
| ( ) Space coordinate system | ( ) Represent geometric objects with coordinates |
| ( ) Space vector | ( ) Coordinate representation of vectors |
| ( ) Represent geometric propositions with vectors | ( ) Select geometric problem-solving strategies |
| ( ) Geometric modeling | ( ) Geometric deductive reasoning |

4. Does “Define solids” require the following knowledge and skills?

(Check the options that you think are required)

| ( ) Spatial Visualization | ( ) Recognize solids |
| --- | --- |
| ( ) Classify solids | ( ) Geometric drawing |
| ( ) Geometric positional relationships | ( ) Represent geometric propositions |
| ( ) Axiomatic system | ( ) Use geometric postulates |
| ( ) Space coordinate system | ( ) Represent geometric objects with coordinates |
| ( ) Space vector | ( ) Coordinate representation of vectors |
| ( ) Represent geometric propositions with vectors | ( ) Select geometric problem-solving strategies |
| ( ) Geometric modeling | ( ) Geometric deductive reasoning |

5. Does “Classify solids” require the following knowledge and skills?

(Check the options that you think are required)

| ( ) Spatial Visualization | ( ) Recognize solids |
| --- | --- |
| ( ) Define solids | ( ) Geometric drawing |
| ( ) Geometric positional relationships | ( ) Represent geometric propositions |
| ( ) Axiomatic system | ( ) Use geometric postulates |
| ( ) Space coordinate system | ( ) Represent geometric objects with coordinates |
| ( ) Space vector | ( ) Coordinate representation of vectors |
| ( ) Represent geometric propositions with vectors | ( ) Select geometric problem-solving strategies |
| ( ) Geometric modeling | ( ) Geometric deductive reasoning |

6. Does “Geometric drawing” require the following knowledge and skills?

(Check the options that you think are required)

| ( ) Spatial Visualization | ( ) Recognize solids |
| --- | --- |
| ( ) Define solids | ( ) Classify solids |
| ( ) Geometric positional relationships | ( ) Represent geometric propositions |
| ( ) Axiomatic system | ( ) Use geometric postulates |
| ( ) Space coordinate system | ( ) Represent geometric objects with coordinates |
| ( ) Space vector | ( ) Coordinate representation of vectors |
| ( ) Represent geometric propositions with vectors | ( ) Select geometric problem-solving strategies |
| ( ) Geometric modeling | ( ) Geometric deductive reasoning |

7. Does “Geometric positional relationships” require the following knowledge and skills?

(Check the options that you think are required)

| ( ) Spatial Visualization | ( ) Recognize solids |
| --- | --- |
| ( ) Define solids | ( ) Classify solids |
| ( ) Geometric drawing | ( ) Represent geometric propositions |
| ( ) Axiomatic system | ( ) Use geometric postulates |
| ( ) Space coordinate system | ( ) Represent geometric objects with coordinates |
| ( ) Space vector | ( ) Coordinate representation of vectors |
| ( ) Represent geometric propositions with vectors | ( ) Select geometric problem-solving strategies |
| ( ) Geometric modeling | ( ) Geometric deductive reasoning |

8. Does “Represent geometric propositions” require the following knowledge and skills?

(Check the options that you think are required)

| ( ) Spatial Visualization | ( ) Recognize solids |
| --- | --- |
| ( ) Define solids | ( ) Classify solids |
| ( ) Geometric drawing | ( ) Geometric positional relationships |
| ( ) Axiomatic system | ( ) Use geometric postulates |
| ( ) Space coordinate system | ( ) Represent geometric objects with coordinates |
| ( ) Space vector | ( ) Coordinate representation of vectors |
| ( ) Represent geometric propositions with vectors | ( ) Select geometric problem-solving strategies |
| ( ) Geometric modeling | ( ) Geometric deductive reasoning |

9. Does “Axiomatic system” require the following knowledge and skills?

(Check the options that you think are required)

| ( ) Spatial Visualization | ( ) Recognize solids |
| --- | --- |
| ( ) Define solids | ( ) Classify solids |
| ( ) Geometric drawing | ( ) Geometric positional relationships |
| ( ) Represent geometric propositions | ( ) Use geometric postulates |
| ( ) Space coordinate system | ( ) Represent geometric objects with coordinates |
| ( ) Space vector | ( ) Coordinate representation of vectors |
| ( ) Represent geometric propositions with vectors | ( ) Select geometric problem-solving strategies |
| ( ) Geometric modeling | ( ) Geometric deductive reasoning |

10. Does “Use geometric postulates” require the following knowledge and skills?

(Check the options that you think are required)

| ( ) Spatial Visualization | ( ) Recognize solids |
| --- | --- |
| ( ) Define solids | ( ) Classify solids |
| ( ) Geometric drawing | ( ) Geometric positional relationships |
| ( ) Represent geometric propositions | ( ) Axiomatic system |
| ( ) Space coordinate system | ( ) Represent geometric objects with coordinates |
| ( ) Space vector | ( ) Coordinate representation of vectors |
| ( ) Represent geometric propositions with vectors | ( ) Select geometric problem-solving strategies |
| ( ) Geometric modeling | ( ) Geometric deductive reasoning |

11. Does “Space coordinate system” require the following knowledge and skills?

(Check the options that you think are required)

| ( ) Spatial Visualization | ( ) Recognize solids |
| --- | --- |
| ( ) Define solids | ( ) Classify solids |
| ( ) Geometric drawing | ( ) Geometric positional relationships |
| ( ) Represent geometric propositions | ( ) Axiomatic system |
| ( ) Use geometric postulates | ( ) Represent geometric objects with coordinates |
| ( ) Space vector | ( ) Coordinate representation of vectors |
| ( ) Represent geometric propositions with vectors | ( ) Select geometric problem-solving strategies |
| ( ) Geometric modeling | ( ) Geometric deductive reasoning |

12. Does “Represent geometric objects with coordinates” require the following knowledge and skills?

(Check the options that you think are required)

| ( ) Spatial Visualization | ( ) Recognize solids |
| --- | --- |
| ( ) Define solids | ( ) Classify solids |
| ( ) Geometric drawing | ( ) Geometric positional relationships |
| ( ) Represent geometric propositions | ( ) Axiomatic system |
| ( ) Use geometric postulates | ( ) Space coordinate system |
| ( ) Space vector | ( ) Coordinate representation of vectors |
| ( ) Represent geometric propositions with vectors | ( ) Select geometric problem-solving strategies |
| ( ) Geometric modeling | ( ) Geometric deductive reasoning |

13. Does “Space vector” require the following knowledge and skills?

(Check the options that you think are required)

| ( ) Spatial Visualization | ( ) Recognize solids |
| --- | --- |
| ( ) Define solids | ( ) Classify solids |
| ( ) Geometric drawing | ( ) Geometric positional relationships |
| ( ) Represent geometric propositions | ( ) Axiomatic system |
| ( ) Use geometric postulates | ( ) Space coordinate system |
| ( ) Represent geometric objects with coordinates | ( ) Coordinate representation of vectors |
| ( ) Represent geometric propositions with vectors | ( ) Select geometric problem-solving strategies |
| ( ) Geometric modeling | ( ) Geometric deductive reasoning |

14. Does “Coordinate representation of vectors” require the following knowledge and skills?

(Check the options that you think are required)

| ( ) Spatial Visualization | ( ) Recognize solids |
| --- | --- |
| ( ) Define solids | ( ) Classify solids |
| ( ) Geometric drawing | ( ) Geometric positional relationships |
| ( ) Represent geometric propositions | ( ) Axiomatic system |
| ( ) Use geometric postulates | ( ) Space coordinate system |
| ( ) Represent geometric objects with coordinates | ( ) Space vector |
| ( ) Represent geometric propositions with vectors | ( ) Select geometric problem-solving strategies |
| ( ) Geometric modeling | ( ) Geometric deductive reasoning |

15. Does “Represent geometric propositions with vectors” require the following knowledge and skills?

(Check the options that you think are required)

| ( ) Spatial Visualization | ( ) Recognize solids |
| --- | --- |
| ( ) Define solids | ( ) Classify solids |
| ( ) Geometric drawing | ( ) Geometric positional relationships |
| ( ) Represent geometric propositions | ( ) Axiomatic system |
| ( ) Use geometric postulates | ( ) Space coordinate system |
| ( ) Represent geometric objects with coordinates | ( ) Space vector |
| ( ) Coordinate representation of vectors | ( ) Select geometric problem-solving strategies |
| ( ) Geometric modeling | ( ) Geometric deductive reasoning |

16. Does “Select geometric problem-solving strategies” require the following knowledge and skills?

(Check the options that you think are required)

| ( ) Spatial Visualization | ( ) Recognize solids |
| --- | --- |
| ( ) Define solids | ( ) Classify solids |
| ( ) Geometric drawing | ( ) Geometric positional relationships |
| ( ) Represent geometric propositions | ( ) Axiomatic system |
| ( ) Use geometric postulates | ( ) Space coordinate system |
| ( ) Represent geometric objects with coordinates | ( ) Space vector |
| ( ) Coordinate representation of vectors | ( ) Represent geometric propositions with vectors |
| ( ) Geometric modeling | ( ) Geometric deductive reasoning |

17. Does “Geometric modeling” require the following knowledge and skills?

(Check the options that you think are required)

| ( ) Spatial Visualization | ( ) Recognize solids |
| --- | --- |
| ( ) Define solids | ( ) Classify solids |
| ( ) Geometric drawing | ( ) Geometric positional relationships |
| ( ) Represent geometric propositions | ( ) Axiomatic system |
| ( ) Use geometric postulates | ( ) Space coordinate system |
| ( ) Represent geometric objects with coordinates | ( ) Space vector |
| ( ) Coordinate representation of vectors | ( ) Represent geometric propositions with vectors |
| ( ) Select geometric problem-solving strategies | ( ) Geometric deductive reasoning |

18. Does “Geometric deductive reasoning” require the following knowledge and skills?

(Check the options that you think are required)

| ( ) Spatial Visualization | ( ) Recognize solids |
| --- | --- |
| ( ) Define solids | ( ) Classify solids |
| ( ) Geometric drawing | ( ) Geometric positional relationships |
| ( ) Represent geometric propositions | ( ) Axiomatic system |
| ( ) Use geometric postulates | ( ) Space coordinate system |
| ( ) Represent geometric objects with coordinates | ( ) Space vector |
| ( ) Coordinate representation of vectors | ( ) Represent geometric propositions with vectors |
| ( ) Select geometric problem-solving strategies | ( ) Geometric modeling |
